# Primary erythromelalgia mainly manifested by hypertensive crisis: A case report and literature review

**DOI:** 10.3389/fped.2022.796149

**Published:** 2022-08-16

**Authors:** Shuo Feng, Zhanwen He, Liping Que, Xiangyang Luo, Liyang Liang, Dongfang Li, Lijun Qin

**Affiliations:** Sun Yat-sen Memorial Hospital, Sun Yat-sen University, Guangzhou, China

**Keywords:** nitroglycerin, genetic diagnosis, pain, bradycardia, erythromelalgia

## Abstract

**Background:**

Primary erythrocytic (PEM) is a rare autosomal dominant single gene disease. Most of the changes of gene loci can be found by whole exon gene sequencing, and the clinical symptoms and patient survival can be improved by specific site-to-site drug treatment. The other manifestations of this patient population are not remarkable. After the application of common drugs, the toxicity and side effects can be limiting. In addition to other common clinical manifestations, we found that the only unique manifestation of this patient was hypertensive crisis. Following multidisciplinary diagnosis and treatment (MDT), we decided to first control hypertension to alleviate the acute and critical patients. However, after controlling the hypertensive crisis, we unexpectedly found that the clinical symptoms of the patients had been significantly improved. Therefore, we concluded that the use of antihypertensive drugs can treat erythematous limb pain with the clinical manifestation of hypertensive crisis. Here, we describe a typical PEM disease, primary clinical features, diagnosis and treatment.

**Methods:**

Medical records of an 8-year-old boy with PEM were analyzed retrospectively, which included clinical characteristics, follow-up information, and SCN9A (Sodium Voltage-Gated Channel Alpha Subunit 9) gene analysis.

**Results:**

The 8-year-old boy had complained of abnormal paresthesia in his feet and ankles with burning sensation and pain for 2 years. The skin of both lower legs was red and underwent ichthyosis and lichenification. Genetic analysis confirmed the existence of a SCN9A gene mutation. The symptoms were gradually improved by treating with intravenous drip and oral administration of nitroglycerin to slow his heart rhythm.

**Conclusion:**

Primary erythrocytic is characterized by skin ulceration, redness, elevated temperature, and severe burning pain primarily in both lower extremities. PEM can be diagnosed by genetic analysis. As this case demonstrates, treating with nitroglycerin as the drug of choice to control the hypertensive crisis significantly improved the symptoms of PEM and hypertension in this patient.

## Introduction

Erythromelalgia (EM) is a rare autosomal dominant single-gene genetic disorder mainly characterized by burning-pain in the extremities and changes of skin color and structure. Laboratory tests shows that muscle injury in certain patients, with slight increase of alanine aminotransferase, aspartate aminotransferase and erythrocyte sedimentation rate. Pathologically, some skin injuries are associated with nerve and fiber involvement. In the field of epidemiology, erythrocyte pain may develop from either primary, resulting from myeloproliferative disorder or secondary, resulting from a hemoglobinopathy, chronic hypoxia, malignancy, or dysregulated erythropoietin production. Primary EM (PEM) is more common in children and adolescents. In some patients, a positive family history may clearly show an autosomal dominant inheritance pattern. Herein, we describe a typical case of PEM. The child has only preliminary skin changes. Due to timely treatment and intervention, the patient has no nerve and fiber damage. Clinical diagnosis for EM is often earlier than genetic diagnosis, leading to block the EM progress but limit, accuracy. Whole exome gene analysis may provide the most accurate genetic diagnosis for EM.

## Case description

Clinical characteristics: An 8-year-old boy from Chenzhou City, Hunan Province, China visited Sun Yat-sen Memorial Hospital of Sun Yat-sen University, China on October 29, 2020 for the first time with complaints of abnormal sensation in both feet over the past 2 years. The burning sensation and mild itching symptoms in both feet and ankles started with no obvious cause. The symptoms were not relieved by treating with topical glucocorticoids but were alleviated by soaking in iced water. Six months ago, the symptoms became worse, spreading from the ankles of the feet to the lower extremities, accompanied by burning pain, severe itching but normal skin temperature, and scattered chilblain skin lesions on both lower extremities. The patient sought the local clinics several times where he was diagnosed with allergic skin rash and treated with dioxypromazine granules, zinc oxide borate borneol ointment and naftifine ketoconazole cream. However, these treatments did not relieve the symptoms.

The patient was born prematurely at G2P2 (Gravida 2 and Pregnancies 2) and lagged behind his peers the same age in growth and development. The patient had difficulty in socialization with peers due to skin lesions on both lower extremities.

**FIGURE 1:**
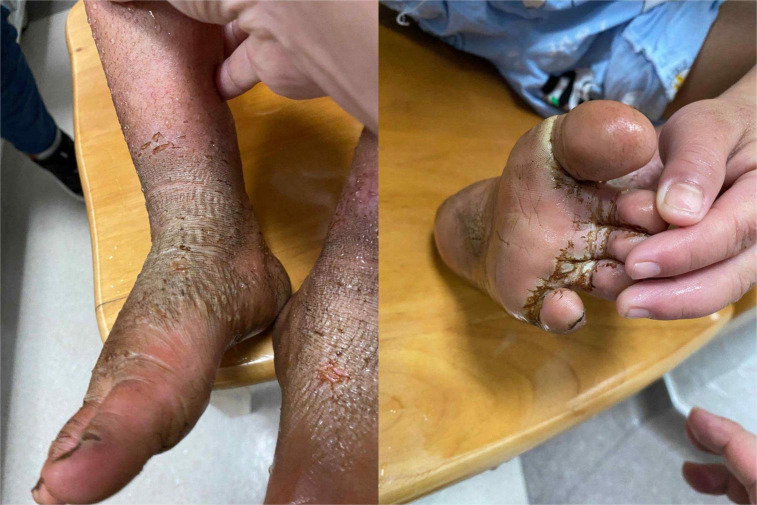
The skin of both soles and heels and between the toes was chapped and ulcerated to form frostbite-like skin lesions.

**FIGURE 2:**
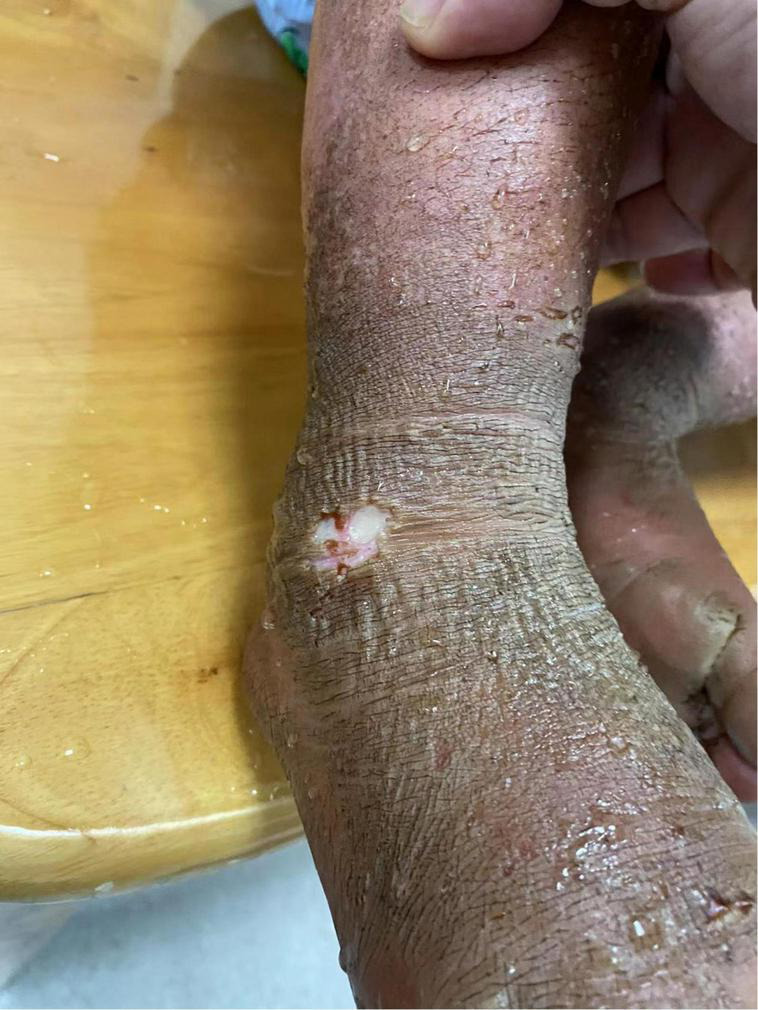
Two ulcer lesions were observed on the skin of the right ankle.

Physical examination: Normal skin temperature and ruddy skin in both lower legs with ichthyosis and lichenification, height 108 cm (<−3 SD), weight 15 kg (<−3 SD) blood pressure 148/100 mmHg, normal heart rhythm, and heart rate 156 beats/min. The patient was poorly developed and malnourished with unintentional weight loss. Skin redness and swelling on both lower extremities were visible, with focus on the ankles and feet accompanied ichthyosis and lichenification. The skin of both soles and heels and between the toes was chapped and ulcerated to form frostbite-like skin lesions (Figure 1). Two ulcer lesions were observed on the skin of the right ankle (Figure 2).

Laboratory examination: blood routine and biochemical tests were performed. White blood cell count was 13.67 × 109/L (Normal: 4.0–10.0 × 10^9^ g/L); Red blood cell count was 4.33 × 1012/L (Normal: 4.2–6.0 × 10^12^ g/L); Platelet count was 474 × 109/L (Normal: 125–350 × 10^9^ g/L); Neutrophils were 10.61 × 109/L (Normal: 1.8–6.3 × 10^9^ g/L). Blood glucose was within the normal range. Creatine kinase was 1,662 U/L (Normal: 26–174 U/L); lactate dehydrogenase was 597 U/L (Normal: 108–252 U/L); creatine kinase isoenzyme was 103 U/L (Normal: 0–24 U/L); and uric acid was 508 μmol/L (Normal: 120–450 μmol/L). Antinuclear antibody (ANA), rheumatoid factor (RF), and antistreptolysin O (ASO) screening data were within the normal ranges.

Pathological examination: In view of this specific dermal change, we performed a skin pathological examination of the subject after admission. The pathological examination showed only mild hypertrophy of the epidermis with hyperkeratosis and mild thickening of the granular layer in the skin tissue. No obvious abnormalities in the blood vessels or nerves in the dermis or subcutaneous fat were observed.

Taken together, the patient was diagnosed PEM. SCN9A genetic testing was performed in the patient and his parents to assist in understanding the patient’s disorder. Follow-up genetic verification for SCN9A was performed in his sister.

## Results

### Genetic analysis of SCN9A gene

**FIGURE 3:**
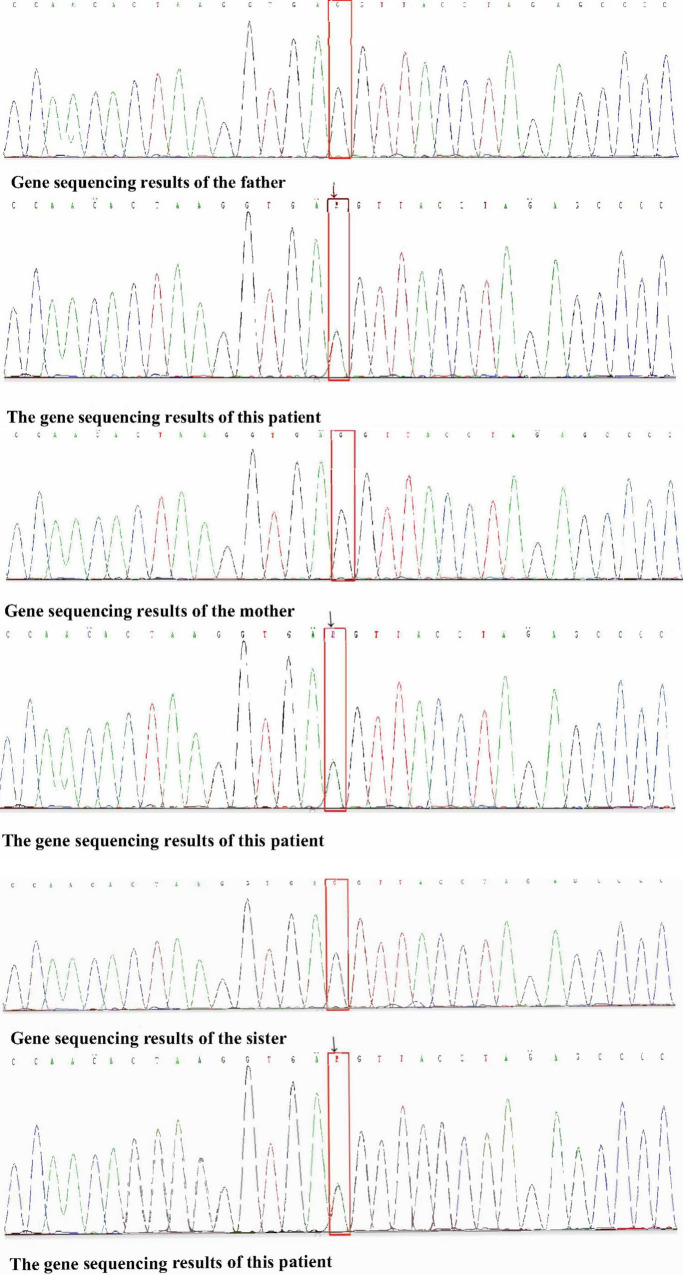
Sequencing map of gene loci between father/mother/sister and child.

Whole-exon gene sequencing analysis identified missense mutations in the SCN9A gene [p. Leu 869Phe (heterozygous), c.2605C > T]. Genetic analysis of the SCN9A gene showed no mutation in his sister. Therefore, the patient was diagnosed PEM. The whole exon genetic sequencing of the child’s family pedigree suggested obvious SCN9A gene defects only in the child. The child’s father, mother and sister showed no similar genetic defects (Figure 3).

### Treatment and outcome

**FIGURE 4:**
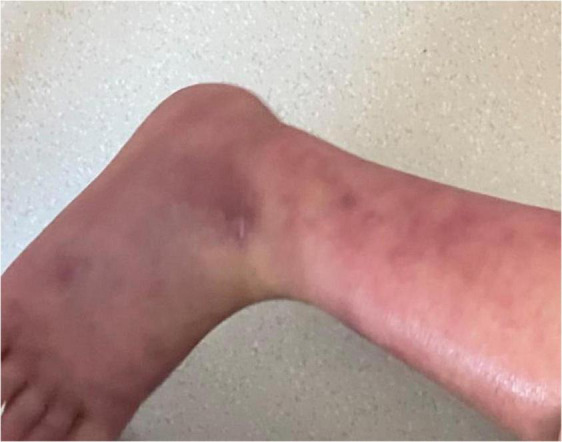
Post-discharge skin changes in children.

**FIGURE 5:**
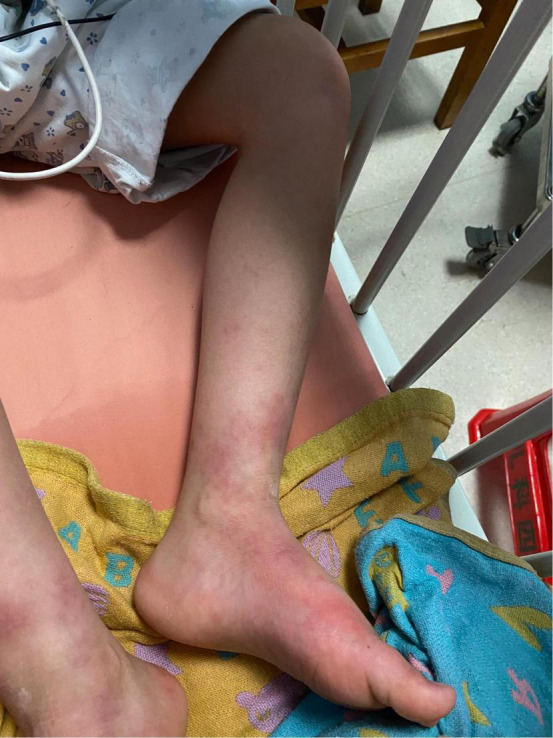
Follow-up of the patient out of hospital -3months later.

**FIGURE 6:**
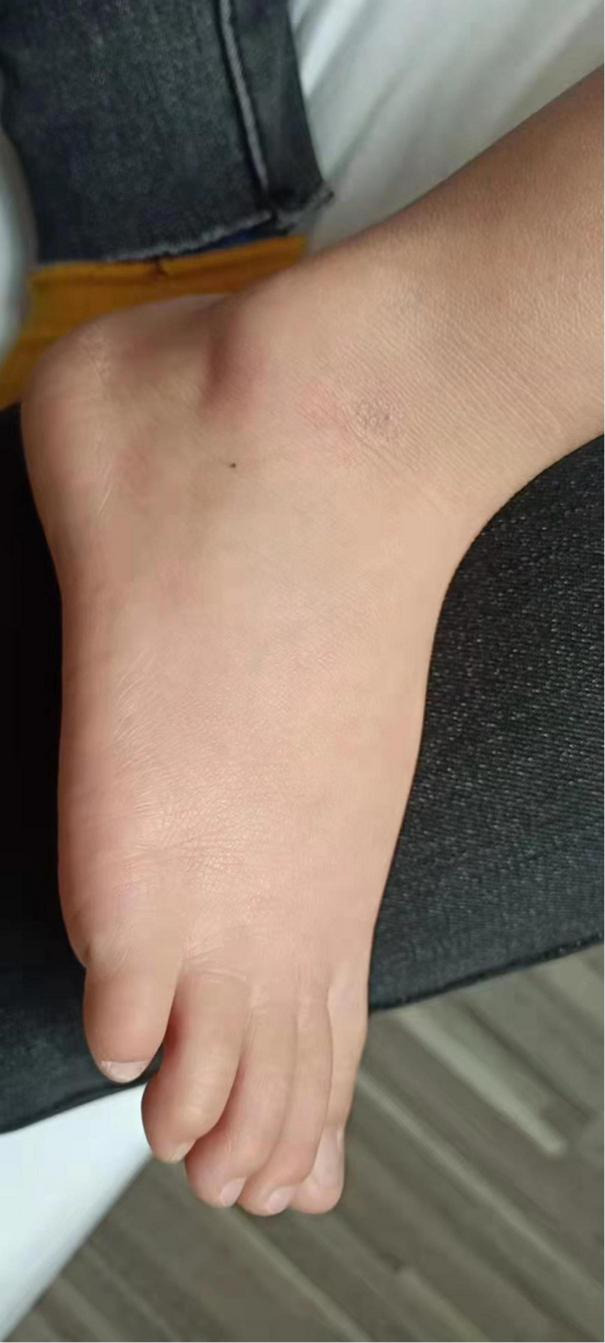
Follow-up of the patient out of hospital -6months later.

**FIGURE 7:**
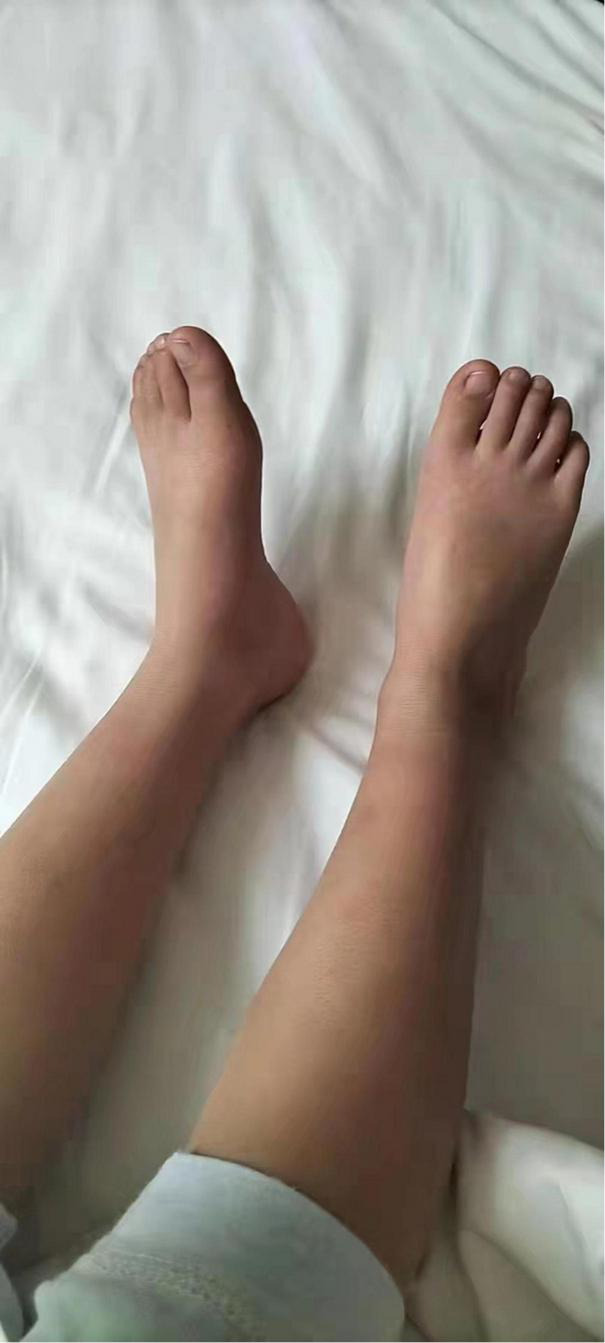
Follow-up of the patient out of hospital -1year later.

We initially treated patients with anti-inflammatory methylprednisolone (2 mg/kg 5 days, 1.5 mg/kg 2 days, and 1 mg/kg 3 days), desloratadine to improve allergic symptoms (2.5 mg quarter night), dipyridamole tablets to prevent platelet aggregation (12.5 mg three times a day) and human immunoglobulin to improve immunity (2 G/kg). After 1 week of treatment, the symptoms had not been significantly improved. However, the patient’s blood pressure increased to 160/120 mmHg (>NHBPEP 99th). In order to improve the hypertensive crisis and prevent the risk of brain hemorrhage, we gave the patient nifedipine (4 mg), but the patient still had hypertension. Subsequently, we injected nitroglycerin (1 μg/kg. Min) intravenously to reduce the blood pressure to 104–130/73–113 mmHg (NHBPEP 95th–99th). Nitroglycerin can significantly improve the skin paresthesia, lesions and color of both lower limbs, and reduce pain. After blood pressure was stabilized, the patient took 25 mg of mexiletine (an antiarrhythmic drug that inhibits sodium influx into cardiomyocytes) orally every day. Compared with nitroglycerin, oral mexiletine for 7 days did not significantly improve symptoms. After the hypertensive crisis was controlled, the oral dose of mexiletine was increased to 100 mg, and Betaloc (25 mg) and fosinopril sodium (5 mg) were supplemented daily to control blood pressure. Subsequently, the dose of mexiletine was gradually increased to 150 mg per day for 1 month. One month later, the pain of both lower limbs was significantly relieved. During follow-up, we found that the patient had congenital heart disease in addition to PEM. Interventional closure was performed after the patient was fasted routinely and stopped medication. During this period, the patient did not take drugs regularly due to routine fasting and drinking before the index procedure, and had never had hypertensive crisis. Blood pressure was as high as 168/100 mmHg, resulting in redder skin and aggravation of lower limb pain. After the operation (CHD-Interventional closure of congenital heart disease), the patient continued to use nitroglycerin intravenous pump (1.5 μg/kg. Min) to control the blood pressure at 110/86 mmHg (NHBPEP 90th), to improve skin injury and color of both lower limbs. The patient was discharged after his blood pressure stabilized. The patient was discharged after his blood pressure stabilized and the skin damage was relieved (Figure 4). After discharge from the hospital, the subject was followed-up for 1-year, and showed recovery of the skin to normal (Figures 5–7).

## Discussion

Erythromelalgia was first diagnosed in Kvernebo Weir Mitchell, and officially named by Smith and Allen to emphasize the characteristic warmth of this syndrome. Babb et al. classified erythromelalgia as primary (PEM) or secondary (1, 2). Among them, most of the PEM are rare autosomal dominant disorders mainly caused by abnormalities of the voltage-gated sodium channel α-subunit Nav 1.7 encoding gene (SCN9A) gene. The incidence rate of PEM is relatively high in children and adolescents. Secondary erythromelalgia is more common in autoimmune and myelodysplastic diseases, and more prevalent in adults, especially in patients with autoimmune deficiency (3), but rare after surgery (4). The main clinical characteristics of PEM are skin swelling, increased temperature and burning pain primarily in the lower extremities. Elevated environmental temperature, trauma, long-term migration, and long-term heat will aggravate or relieve pain. Skin ulcers and infections are common complications. Severe cases can lead to gangrene, amputation, nail growth disorders, and even fatal hypothermic necrosis. As this case demonstrates, an 8-year-old boy claimed abnormal paresthesia in feet and ankles with burning sensation and pain for 2 years. The main symptoms had been aggravated during the 6-months prior to seeking care at Sun Yat-sen Memorial Hospital. The skin of both lower legs was redness and underwent ichthyosis and lichenification. The blood pressure was within normal range. The patient had no history of taking special drugs. Laboratory tests excluded autoimmune diseases and hematological malignancies. Therefore, the patient was clinically diagnosed with PEM.

The pathogenesis of PEM was previously believed to be associated with autonomic and vascular dysfunction that caused uneven distribution of nutrients and insufficient capillary perfusion in the skin and capillaries of the affected area. Nervous system stimulation causes continuous tissue hypoxia and dilation of arteries. Low temperature has been shown to alleviate symptoms by reducing the congestion and perfusion of the damaged skin (5).

The pathophysiology of hypertension is still not well understood. The level of arterial pressure depends on the stroke volume and total peripheral vascular resistance. Any factor that causes stroke volume to increase, such as elevating blood volume and myocardial contractility after water and sodium retention or increasing peripheral vascular resistance by nerve or endocrine factors that cause peripheral arterial contraction, will elevate blood pressure. The former increases systolic blood pressure, while the latter increases diastolic blood pressure. Skin damage of the lower extremities in this patient was caused by continuous hypoxia of the tissue and the dilation of the arteries, which might be related to the increase of peripheral vascular resistance (5).

There is no standard pathological diagnosis for erythromelalgia (5). Diagnosis of erythromelalgia is based on clinical criteria and exclusion of differential diagnoses, including peripheral neuropathies, vasculitis, acrocyanosis, and Fabry disease. Capillary proliferation, a large number of vascular nests, vascular endothelial cell damage, perivascular inflammatory changes, and deposition of immune complexes including C3 and fibrin were observed in skin biopsy. Biopsy in patients with PEM is non-specific and is not considered useful for diagnosis or treatment decisions (5). With the development of molecular medical diagnosis, gene sequencing has become the gold standard for diagnosis of erythromelalgia. Whole exome gene sequencing analysis revealed an association between SCN9A gene with the Nav1.7 sodium ion channel.

The SCN9A gene encodes a sodium ion channel (VGSC) protein subtype Nav1.7 and plays an important physiological function in cell production, transmission, and regulation. Increasing cell proliferation and current U-turn time can increase neuronal sensitivity, cause slight irritation and reduce the constant visual range of the relationship, resulting in long-term severe pain (6). Mutations in sodium channels also affect vascular regulation, leading to elevated blood flow and skin temperature. Over 19 mutations in the Nav1.7 sites have been characterized, including F216S, S241T, N395K, I848T, L858F, L858H, A863P, F1449V, I136IV, P610T, L823R, S459X, I767X, W897, L1331P, M1627K, I1461T, T132E464I, and A1627K (7). Genetic testing revealed that the patient in the present study had a new mutation site on chr2: 167 133762 (c.2605C > T) that was already collected in the ClinVar database (Variation ID: 6364PEM) and is caused by an autosomal dominant mutation. Therefore, an affected individual will have 50% chance of passing on the mutation to each offspring. Retrospective analysis of the patient and his elder sister and parents showed no obvious family history. Therefore, it is necessary to further clarify the correlation between the mutation of this gene and the phenotype.

Nav1.7 is preferentially expressed in many tissues, including dorsal root ganglia (DRG), trigeminal ganglia, olfactory epithelium and sympathetic neurons. Recently, Nav1.7 was reported to be expressed in the hypothalamus and free nerve endings of the superficial plate of the spinal dorsal horn. Functional mutations of the SCN9A genes are associated with many genetic diseases, including congenital pain insensitivity, primary limb pain erythema, paroxysmal extreme pain disorder, and small fiber neuropathy (7). Effective Nav1.7 blockers lack effective analgesia, indicating that in addition to electrical conduction, the Nav1.7 channel is also involved more complex pain processes. In fact, recent studies showed that the deletion of Nav1.7 in mouse DRG sensory neurons caused disorders of a series of genes (194 genes > 1.5-fold disorders), including upregulation of a gene encoding enkephalin precursor Penk (A well-defined endogenous opioid) (8, 9). In addition, the endogenous analgesic effect was reversed by injecting naloxone into human congenital insensitivity to pain (CIP) individuals with two SCN9A mutations and mice without nav1.7 mutations. Changes in the expression of endogenous opioid peptides explained the CIP phenotype of Nav1.7 deletion mutant mice and non-functional Nav1.7 in humans, suggesting a potential link between sodium interaction through Nav1.7 and transcriptional regulation. In fact, the link between transcription and ion channels has been well proven (10, 11) in closely related voltage-gated sodium channel (VGSCs). The multiple functions of Nav1.7 explain insufficient effectiveness of Nav1.7 blockers (12). Combination of therapies targeting Nav1.7 with sodium channel blockers and opioids appears to be a potentially more effective analgesic strategy.

Based on clinical criteria and the presence of mutations in the SCN9A gene, the patient was diagnosed with PEM. Treatment of PEM is primarily focused on symptom control with drugs and surgery. Drug treatments include the more commonly used oral neuropathic medications, intravenous infusions, and invasive procedures such as non-steroidal anti-inflammatory drugs, antidepressants, anticonvulsants, antihistamines, vasodilators, sodium ion channels blockers and prostaglandins. The occurrence of erythromelalgia is related to the function-enhancing mutation of the NaV1.7 sodium channel gene SCN9A, therefore, sodium channel blockers are the most common pharmacotherapy targeting PEM (13). Among them, class IB antiarrhythmic drugs lidocaine and mexiletine are most commonly used at the therapeutic dose of 16.5 μg/(kg min; iv). The oral administration dose of slow heart rhythm was maximally 200 mg three times a day. Treatment with slow heart rhythm can be very effective for PEM. The symptoms in most patients can be relieved after 2 weeks of treatment with slow heart rhythm. The maximum duration of use can be over a period of up to 6 months without increasing the dose (14, 15). As this case demonstrates, the slow heart rate was increased to 175 mg per day at 1 month until the follow-up visit, leading to significant alleviation of the skin lesions and pain of both lower limbs.

Non-steroidal anti-inflammatory drugs usually have antipyretic and analgesic effects. Among them, aspirin is the most commonly used, which can not only relieve fever and analgesia, but also has anti-platelet aggregation and thrombosis. Oral administration dose of aspirin is 15 mg/(kg day) (15). As this case demonstrates, we treated the patient with 50 mg of aspirin per day for 7 weeks, and scheduled him for congenital heart disease interventional surgery while continuing to administer a higher dose of aspirin (150 mg/day) after the operation. The patient remains under follow-up.

Antispasmodics include carbamazepine and gabapentin. Carbamazepine can cause the loss of the sodium ion voltage network and reduce the sensitivity of nerve nodes (12). Gabapentin is connected to the L-calcium ion input channel. The combination of these two drugs is more effective than either of the single drugs alone. The regimen consists of carbamazepine at 300 mg two times per day with gabapentin titrated up to 300 mg five times per day. The effect of combined treatment typically occurs within 1 week and lasts for more than 1 year without obvious adverse reactions (16).

Vasodilator sodium nitroprusside has been used to quickly relax vascular smooth muscle, reduce peripheral vascular resistance, and dilate blood vessels. The mechanism of vasodilator sodium nitroprusside in the treatment of PEM is not clearly understood. The initial dose is commonly 0.5 μg/(kg min), and the maximum dose is 5 μg/(kg min). Vasodilator sodium nitroprusside has adverse reactions such as hypotension and acidosis and requires physician supervision for long-term application (14). Nitroglycerin has the same effect as nitroprusside. As this case demonstrates, the skin lesions and pain of both lower limbs were significantly improved after administering nitroglycerin. In addition to alleviating the symptoms of PEM, nitroglycerin also alleviates hypertensive crisis.

Surgical treatment options include epidural block, sympathetic ganglion block (13), sympathectomy, and brachial plexus block. Since the neuropathic pain of erythromelalgia is mainly caused by spinal ganglia, sensory ganglia and sensory nerve sodium ion transport, the surgical operation to inject certain drugs such as narcotic drugs, opioids and medicinal hormones into the diseased area near the nerve center may alleviate symptoms through interruption in nerve sodium ion transport. These narcotic drugs can stabilize the membrane potential of nerve cells and the permeability of the ion membrane to reduce sodium hormone leading to anti-inflammatory effects. Electrophysiology is more commonly used to change neuromuscular contact and nerve conduction, and cause anesthesia by inhibiting fibers to treat pain. Surgery is a common treatment under clinical conditions for erythromelalgia; however, the effectiveness is variable.

Sympathetic ganglion blocks can either relieve or exacerbate symptoms. Therefore, sympathetic ganglion blocks are usually attempted when other treatments are ineffective. Sympathetic ganglia blocks can improve blood circulation to reduce neurogenic pain and relieve clinical symptoms. As this case demonstrates, since the patient had an obvious hypertensive crisis, and damage to liver and vascular function, he was treated with an initial dose of nitroglycerin (0.1 μg/kg min) to stabilize blood pressure and relieve pain in the lower limbs. The patient still suffered intermittent redness, swelling, heat and pain in both lower limbs after stabilization of blood pressure; therefore, he was administered an initial dose of sodium channel blocker mexiletine (50 mg), which did not significantly relieve symptoms. During treatment, family members of the patient noticed serious side effects of mexiletine and stopped administering mexiletine to the patient, resulting in severe pain in both lower limbs, and blood pressure up to 180/130 mmHg. The patient was again administered nitroglycerinnitric (0.2 μg/kg min) to stablize blood pressure, and the dose of mexiletine was adjusted from 100 to 150 mg, and then to 175 mg, resulting in significant alleviation of the skin color and pain of the lower limbs. Treatment methods are variable and treatment effectiveness cannot be uniformly determined. Sympathetic ganglion blocks can be used when drug treatments are ineffective; however, the actual effectiveness also varies from person to person. Women with a positive family history of erythromelalgia are recommended to undergo prenatal molecular genetic testing for early diagnosis and treatment.

## Author contributions

SF had primary responsibility for the protocol development and patient enrollment. SF and ZH collected the data. SF performed the preliminary data analysis and wrote the manuscript. LpQ, LL, DL, XL, and LjQ assisted in data analysis and critical revision. All authors read and approved the final manuscript and assumed full responsibility for the content of the manuscript.
